# Digital Intervention Strategies for Increasing Physical Activity Among Preschoolers: Systematic Review

**DOI:** 10.2196/28230

**Published:** 2022-01-11

**Authors:** Taren Swindle, Anwesh B Poosala, Nan Zeng, Elisabet Børsheim, Aline Andres, Laura L Bellows

**Affiliations:** 1 Department of Pediatrics University of Arkansas Medical Sciences Little Rock, AR United States; 2 Griffin Hospital Derby, CT United States; 3 Department of Pediatrics University of New Mexico Albuquerque, NM United States; 4 Arkansas Children's Nutrition Center Little Rock, AR United States; 5 Arkansas Children’s Research Institute Little Rock, AR United States; 6 Division of Nutritional Sciences Cornell University Ithaca, NY United States

**Keywords:** physical activity, preschool children, digital, technology, intervention

## Abstract

**Background:**

Digital interventions are increasingly used to improve health behaviors. Improved access and lower costs (relative to in-person interventions) make such interventions appealing. Specifically, digital platforms may be a promising approach for increasing physical activity (PA) in young children.

**Objective:**

The goal of this systematic review was three-pronged: (1) to determine the quality of studies using digital PA intervention strategies with preschool-aged children (ie, 3 to 5 years old); (2) to assess the efficacy of digital interventions and approaches designed to improve PA in preschool-aged children; and (3) to examine theoretical application and implementation outcomes with current approaches to digital PA interventions.

**Methods:**

This review identified and summarized studies on digitally supported interventions for promoting PA in preschool-aged children. We generated 3 lists of relevant search terms that included technology-related terms, PA-related terms, and weight-related terms. The search included Ovid MEDLINE(R) and Epub Ahead of Print, In-Process & Other Non-Indexed Citations, and Daily, Ovid EMBASE, Ovid Cochrane Central Register of Controlled Trials, Ovid Cochrane Database of Systematic Reviews, and Scopus. Study selection was led by a single author and verified by a second; the same 2 authors assessed study quality using a standardized tool, and 3 authors completed data extraction on PA outcomes, theory application, and implementation outcomes.

**Results:**

In total, 601 studies were identified; 8 met the inclusion criteria. For study quality, only 2 studies received an overall rating of strong quality and low risk of bias. All but 1 study had a small sample size (<100). Positive and significant changes in child PA outcomes were reported in only 2 studies with weak overall quality, both of which used child-directed approaches. In total, 5 studies applied a behavioral theory for designing the intervention; no patterns of effectiveness were identified based on the application of theory. Finally, no studies reported on the implementation outcomes of adoption, cost, penetration, or sustainability; 1 study did not assess any implementation outcomes, and no single study reported on more than 2 implementation outcomes. Studies measured the implementation outcome of acceptability most frequently (n=4), and researchers assessed fidelity in 3 studies.

**Conclusions:**

The interventions with a significant effect on PA used child-centered activities; parent-directed digital interventions alone were ineffective for improving PA. Future research with rigorous designs, monitoring of implementation outcomes, and testing of the contributions of digital components will advance understanding of the effectiveness of digital interventions for increasing PA in children.

## Introduction

Pediatric obesity is a major global health challenge jeopardizing development and well-being even beyond childhood [1-3]. Physical inactivity and sedentary lifestyle behaviors can contribute to childhood obesity. Regular physical activity (PA) in early life promotes healthy growth and development, improves children’s’ cardiovascular fitness, and promotes better motor and cognitive skill development [4]. Further, active children are less prone to develop chronic diseases later in life including cardiovascular diseases, type II diabetes mellitus, and obesity, as well as psychiatric, psychological, and psychosocial disorders when compared to inactive children [5-7].

Guidelines implemented in the United States for PA in preschool-aged children (3 to 5 years) encourage children to be active throughout the day by engaging in a variety of activities [8]. Although no specific amount is recommended, a reasonable target of 3 hours of movement—with 60 minutes of moderate to vigorous physical activity (MVPA)— is in line with the guidelines from the American Medical Association and the World Health Association [8,9]. Unfortunately, a significant proportion of preschool-aged children do not meet this target (less than 50% across studies) [7]. Several interventions designed to increase PA or reduce sedentary behaviors (eg, limit screen time) have resulted in inconsistent findings [10-12]. According to a meta-analysis of intervention studies with preschool-aged children, only small to moderate effects have been observed for improving PA, suggesting room for improvement in achieving the desired outcomes [13]. Effective PA-promoting interventions targeting preschool children are needed.

The use of newer technologies and digital platforms to mitigate sedentary behaviors and foster behaviors that increase children’s PA may be a promising approach. Digital interventions have become more widely available globally, and health promotion through these platforms has become more accessible, easier to use, and more acceptable to families [14,15]. Indeed, the use of smartphones, websites, and text messaging offer relatively inexpensive and easy solutions to support or replace traditional face-to-face methods [16-19]. Studies with older children, aged 8 to 12 years, have used active video games (ie, exergaming) and digital applications to encourage PA participation [15,20]. Strategies that are child-centered, such as gamification including games with active plots and self-monitoring, have been used in digital applications [18,20]. These studies show that technologies merging PA and learning are available and may be helpful in promoting PA [21]. As the use of digital devices by young children has become common [22], digital platforms may hold significant promise for delivering PA interventions to preschoolers [3,23].

The relatively recent emergence of digital interventions and their potential to create equitable access to PA support also suggests the need to understand factors related to intervention success and failure. The application of behavioral theory for intervention design and implementation outcomes comprises at least 2 such factors. First, the use of theory allows intervention designers to be explicit with the targets for behavior change and select behavior change techniques that affect the theory-informed levers of change [24]. Second, measuring implementation outcomes in the delivery of digital interventions allows researchers to determine (1) if the observed effects (or the lack thereof) were attributable to implementation factors and (2) if the processes or characteristics of the interventions are desirable for larger-scale delivery [25]. Measuring implementation outcomes acknowledges that a potentially effective intervention can be implemented poorly (ie, implementation failure) or an intervention can be ineffective for a new setting (ie, intervention failure) [25]. Thus, assessing theoretical application and implementation outcomes are important for understanding current approaches to digital PA interventions.

There is considerable interest for expanding digital offerings to promote PA in young children, and studies are emerging that leverage digital interventions directed at children and their families. Thus, a review of existing studies to identify common or discriminating features of prior digital interventions contributing to improvements in child PA (or lack thereof) as well as factors associated with successful implementation of digital interventions for activity promotion is warranted. The aim of this systematic review was three-pronged: (1) to determine the quality of studies using digital PA intervention strategies with preschool-aged children; (2) to assess the efficacy of digital interventions and approaches designed to improve PA in preschool-aged children; and (3) to examine theoretical application and implementation outcomes with current approaches to digital PA interventions.

## Methods

The systematic review followed the PRISMA (Preferred Reporting Items for Systematic Reviews and Meta-analyses) reporting guidelines [26].

### Data Sources and Search Strategy

A comprehensive search of English language databases was conducted from each database’s inception to January 24, 2020. The databases included Ovid MEDLINE(R) and Epub Ahead of Print, In-Process & Other Non-Indexed Citations, and Daily, Ovid EMBASE, Ovid Cochrane Central Register of Controlled Trials, Ovid Cochrane Database of Systematic Reviews, and Scopus. The search strategy was designed and executed by an experienced librarian with input from the study's first and second authors (TS and AP). Controlled vocabulary supplemented with keywords was used to search for studies on the efficacy and acceptability of mobile-based, web-based, or other latest technology-supported interventions for promoting PA in preschool-aged children. We supplemented our professional librarian search using the same terms in Google Scholar and by manually searching and reviewing the reference lists of all included articles. The actual strategy listing all search terms used and how they were combined is available in Multimedia Appendix 1.

### Eligibility Criteria

#### Inclusion Criteria

The study designs considered in this review included randomized clinical and controlled trials, and quasi-experimental trials. Studies focusing on promoting PA in typically developing (without systemic, physical, and mental disorders) preschool-aged children (aged 3 to 5 years) were included. Interventions had to engage children or their parents using digitally based modalities to promote PA in children. Studies had to have PA-related outcomes as either the primary or secondary outcome and had to be published in English.

#### Exclusion Criteria

Studies were excluded if the preschool children were not included in the intervention or outcome assessment, if the focus was on health behaviors or conditions other than PA, or if children were not typically developing. Studies were excluded if digitally based platforms were not used as part of the intervention.

### Outcome Variables

The primary outcome variable was PA, including subjectively and objectively determined PA levels (ie, self-reported or observed). PA outcomes included total PA, light physical activity (LPA), MVPA, percentage of time in LPA or MVPA (LPA% or MVPA%), energy expenditure, and steps. When available, weight-related outcomes were extracted as the secondary outcome including, but not limited to, BMI (kg/m^2^), BMI Z-scores (BMIz), body fat (kg), body fat percentage, and waist circumference (cm). Other secondary outcome variables were implementation outcomes based on the taxonomy and definitions for studies reporting on implementation outcomes [25].

### Selection of Articles

The first reviewer (AP) identified duplicates from the searches and screened the titles of the articles to shortlist target articles for review. The second reviewer (NZ) verified the first reviewer’s decisions. The first and second reviewers independently screened the abstracts of the target articles and created a second shortlist. Thereafter, both reviewers read the full texts of the articles independently to evaluate them for inclusion in the final analysis. Discrepancies between the first and second reviewers were discussed and resolved by consensus. When a decision could not be reached, the coauthors reviewed the full texts to make the final decision.

### Assessment of Study Quality and Risk of Bias

Study quality and risk of bias were assessed by 2 independent reviewers (AP and NZ) using the National Collaborating Centre for Methods and Tools Quality assessment tool for quantitative studies [27]. This assessment tool includes ratings (weak, moderate, and strong) for 6 components: (1) selection bias, (2) study design, (3) confounders, (4) blinding, (5) data collection methods, and (6) withdrawals and dropouts. Studies were rated as weak overall if 2 or more components were rated as weak, moderate if only 1 component was rated as weak, and strong if no components were rated as weak. Discrepancies were discussed and resolved by consensus between reviewers to ensure high agreement for ratings. Interrater reliability for individual component ratings was determined by computing the percentage of agreement and the Cohen κ.

### Data Extraction and Synthesis for Study Characteristics, Intervention Efficacy, Use of Theory, and Implementation Outcomes

For primary extraction, each retrieved full-text article was evaluated systematically by 3 reviewers (NZ, AP, and TS) according to the following criteria: (1) design, (2) population, (3) intervention characteristics (modality, ie, how the intervention was delivered, exposure or dose, and content), (4) measures, and (5) analyses and results. As with data extraction, differences were resolved by mutual agreement; all coauthors reviewed the full texts to make the final decision when a decision could not be reached by consensus. TS extracted the theory used in each study, the content of each intervention, and secondary data on implementation, including implementation outcomes, implementation measures, data sources, and results.

## Results

### Selection of Articles

**Figure 1:**
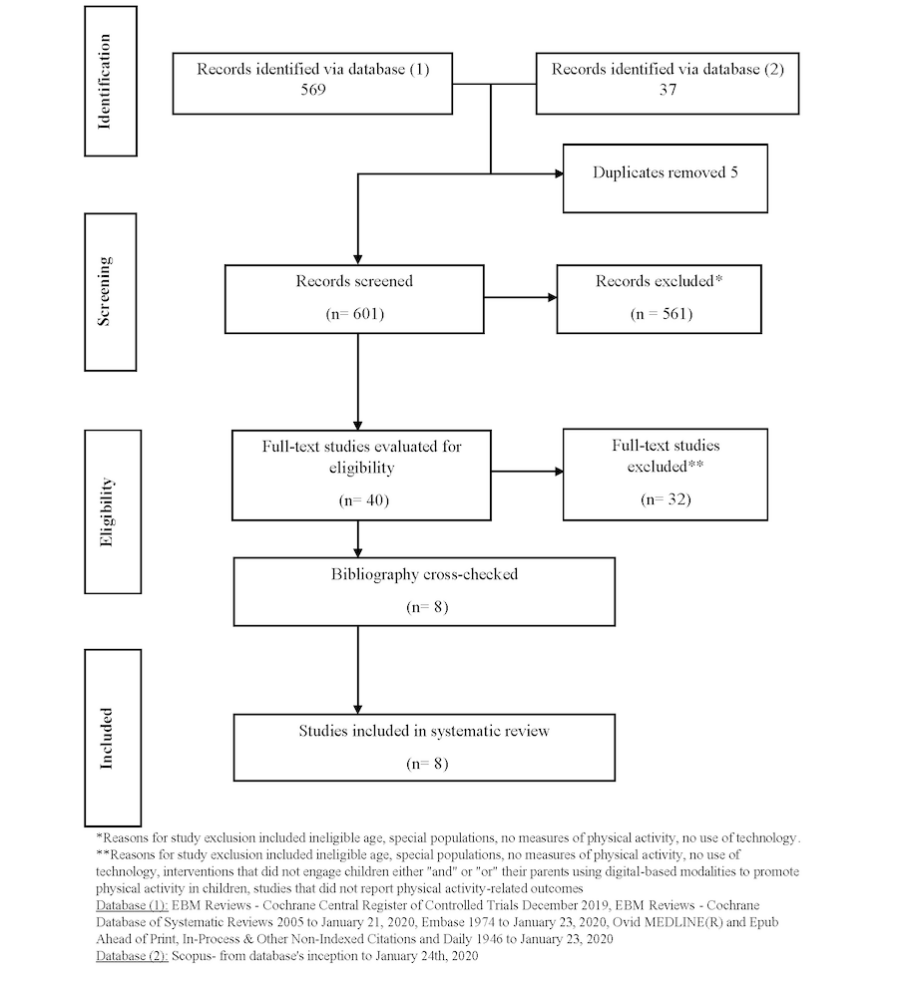
Flow chart for study inclusion.

Figure 1 illustrates the search and selection process for articles. A total of 601 articles were identified initially. After removing duplicates and irrelevant studies, titles and abstracts of the remaining articles were further screened and identified as potentially meeting the inclusion criteria. Following a thorough assessment of the full-text articles, 8 studies fully met the inclusion criteria and were included in this review. The key reasons for excluding articles included ineligible age, special populations, no use of technology, no measure of PA, and non-English articles. A high interrater agreement (> 95%) for inclusion of the articles in this review was obtained between the authors (AP and NZ).

### Assessment of Study Quality and Risk of Bias

Following the ratings of the 6 steps in the National Collaborating Centre for Methods and Quality assessment tool for quantitative studies [27], the design quality and risk of bias for each study are presented in Multimedia Appendix 1. For the initial ratings, the agreement was 93%, and the Cohen κ=0.63. After resolving discrepancies, 2 studies received an overall rating of strong quality and low risk of bias [28,29]; 3 studies received an overall rating of moderate quality and medium risk of bias [30-32]; and 3 studies received an overall rating of weak quality and high risk of bias [33-35]. The most common issues with the study quality and risk of bias were related to selection bias and blinding. More specifically, all studies, apart from 1[31], failed to report the proportion of participants who agreed to participate and were thus unlikely to have a representative sample. Moreover, none of the studies was rated as strong on blinding, as most of the outcome assessors were aware of the research question and the exposure status, a common concern for behavioral intervention studies. Other issues included no reporting of the completion rate, unreliable data collection tools, and failure to control all relevant confounders.

### Study Characteristics

Multimedia Appendix 2 summarizes details of the 8 included studies, ranked by quality. The exposure in most studies was to a technology-based behavior change program, most of which involved PA and nutrition components. All studies were published within the last 6 years (2015-2019) and were conducted in Western countries, with 6 studies conducted in the United States [30-35], 1 in Australia [28], and 1 in Sweden [29]. Of the 8 studies, 5 primarily targeted White samples (41%-100% White) [28,29,31,33,35]; 2 studies targeted samples that were mostly Asian (56% and 100%) [30,32], and 1 study targeted a sample that was mainly Black (42%) [34]. Among these studies, 6 were randomized controlled trials [28-33], and 2 were quasi-experimental trials [34,35]. Of the 8 studies, 3 primarily targeted preschoolers [30,33,35]; 1 had components directed at children and parents [34]; and the remaining 4 studies were aimed at parents [28,29,31,32]. Of the 8 included studies, 2 were feasibility studies with small sample sizes [32,34]. Sample sizes varied across studies, ranging from 32 to 315 participants; 7 studies included <100 participants in total. Control groups or conditions included usual care (eg, Head Start and recess as usual) [30,34,35], free play for children [33], educational resources [28,29,32], and knowledge-based lifestyle programs [31].

Technology modalities in these studies included exergaming (n=3) [30,33,35], social media (Facebook, n=1) with education and social support for parents [35], websites with education modules for parents (n=1) [31], a mobile app with information and strategies for parents (n=1) [29], a preloaded tablet with educational modules for parents (n=1) [32], and a combination of delivery modes including educational and motivational content for parents (eg, emails, social media, online video, telephone calls, and text messages, n=1) [28]. Most studies used a digital platform to deliver health information and education to parents; only the 3 studies evaluating exergaming interventions provided direct PA opportunities to children via a digital modality.

Intervention lengths ranged from 6 weeks to 6 months. Intervention intensity ranged from no required PA sessions to daily 30-minute sessions for 12 weeks. Of the 8 studies, 7 focused on short-term outcomes (≤2 months), and 1 focused on medium-term outcomes (3 to 6 months) [31].

Outcome assessment included objective and subjective measures. In total, 7 studies used objective assessments of PA, including accelerometers and pedometers to capture participants’ (children or parents) PA levels [28-30,33-35]. PA was assessed subjectively in 2 studies [31,32] using parent-reported questionnaires to interpret PA behaviors. All studies assessing weight-related outcomes used objective measures [28-30,32,34]. In addition to PA outcomes, several studies included parent-centered questionnaires related to other targeted behaviors (eg, sedentary behavior, dietary intake, sleep, screen time, feeding practices, parent role modeling, self-efficacy, beverage consumption, and eating behaviors) [28,29,31,32,34]. Child-centered questionnaires were related to enjoyment of movement, perceived competence, and motor skill competence [33,35]. Other objective measures included assessments of cardiovascular fitness, gross motor development, and cognitive flexibility of the children [33,35].

### Intervention Efficacy

#### PA Outcomes

Positive changes in the PA outcomes of children were reported in only 2 studies [33,35]. Fu et al [33] documented an increase of 887 steps via a pedometer among children receiving the intervention compared to the control group, whereas Gao et al [35] demonstrated significant increases in the MVPA time via an accelerometer at the end of the intervention (ie, an increase of 4 minutes per day for the treatment group and a decrease of 2 minutes per day for the control group). Both studies reflect short-term, school-based, child-directed approaches with the intervention centered on providing direct opportunities for PA through exergaming (eg, GoNoodle and Wii Fit).

#### Weight-Related Outcomes

None of the 5 studies examining children’s weight-related outcomes demonstrated significant effects [28-30,32].

#### Other Health Outcomes

Studies produced mixed results on other health-related metrics. In addition to favoring PA outcomes, 2 child-centered (exergaming) interventions reported positive effects on the children’s perceived motor competence [33], gross motor development [33], and cognitive flexibility [30]. In addition, parent-directed interventions produced positive effects for improving self-efficacy [28,31,32] with an exception [34]. No intervention aiming to decrease screen time achieved the desired outcome [28,31,34]. Other outcomes demonstrated no clear patterns of effects.

### Use of Theory and Implementation Outcomes

Multimedia Appendix 3 presents the theory applied by each study and the implementation outcomes assessed. The most commonly used behavioral theory was social cognitive theory (n=3) [28,29,31]. Other theories included the actor-partner interdependence model [34] and the information-motivation-behavior model [32]. Further, 3 studies did not report the explicit use of theories [30,33,35]. No clear patterns of effectiveness were identified based on the application of theory.

No studies reported on the implementation outcomes of adoption, cost, penetration, or sustainability; 1 study did not assess any implementation outcomes [33], and no single study reported on more than 2 implementation outcomes. Studies measured the implementation outcome of acceptability most frequently (n=4), with measures including self-developed surveys [28,34], interviews with participants [32,34], and data from the technology platforms (eg, frequency and duration of use) [31]. The 2 studies with ratings of strong overall quality only examined the implementation outcome of acceptability [28,29]. Researchers assessed fidelity in 3 studies, with 2 monitoring fidelity through observing intervention sessions and completing self-developed checklists [30,35], and 1 study assessed fidelity through a user-completed survey at the end of the intervention [31]. Researchers captured indicators of feasibility and appropriateness in 2 studies. Appropriateness assessments were paired with questions about acceptability in both studies; 1 study focused on the appropriateness of specific intervention aspects (eg, length) through a user-completed survey at the end of the intervention [28], and the other assessed cultural appropriateness in focus groups (preintervention) and interviews (postintervention) [32]. The lack of data on implementation outcomes precludes the ability to link efficacy and implementation outcomes.

## Discussion

### Principal Findings

Prior reviews have shown the value of digital platforms for improving PA-related outcomes in children aged 6 to 12 years [36], adolescents [37], adults [38,39], and older adults [40]; this study sought to determine if similar documented effects exist for children aged 3 to 5 years. Of the 8 studies identified, all were published in the last 6 years with 2 studies showing positive effects on PA in children and only 2 demonstrating a strong quality and low risk of bias (neither of which showed PA effects). Studies measuring implementation outcomes most frequently assessed indicators of acceptability; however, implementation outcomes were not a prominent focus of the studies included. In addition, although most studies applied a theoretical framework, no clear patterns were noted based on use of theory. These findings illustrate the early stages of exploration in this scientific area and the opportunity to conduct future studies that are more rigorous in their design. With such a low number of studies showing changes in activity for children, our ability to examine shared features and patterns was limited.

Based on the limited number of studies showing improved PA in preschool children, common patterns in the effective studies are tentatively noted. Notably, the studies showing effective interventions used child-centered activities (exergaming) in schools; the exergames used in these studies were commercially available (ie, no new intervention or technology was developed). A narrative review noted the strengths of exergaming, including its adaptability, customizability, and scalability for reaching children and adolescents; weaknesses include the lack of sustained engagement over time and costs of development [20]. As technology evolves, it is imperative to understand the attributes of effective child-centered digital activities, such as exergaming, and how other digital platforms (mobile apps, virtual reality, and web-based games) can replicate these outcomes.

Given the limited number of studies in this nascent area of research, this review is more useful to highlight gaps for future work and identify the features of ineffective studies. First, studies aimed at parent education without direct intervention for children did not show desired increases in PA. This finding contradicts a recent review on technology-based and parent-targeted tools for improving nutrition outcomes in children that found positive effects in 10 out of 11 studies [41]. This difference suggests that parents’ gatekeeping behaviors may have a more direct influence on children’s dietary intake than movement habits; engaging parents and children in PA may better support change in this area. Debates about the value of engaging parents versus parent-child dyads or children alone is not new to intervention research. Understanding the effects of the bidirectional relationship between parents and children, particularly the mutual influence of parenting behaviors and children’s outcomes in early childhood, has the potential to enhance the development of digital interventions and their positioning to target audiences [42]. None of the interventions reviewed herein engaged children and parents through the same intervention for mutual increase in PA; this remains an area for future research. Recent formative research suggests that parents are open to the use of digital applications to support such an approach [3].

A second feature of the ineffective studies was the use of subjective measures. Although only 2 studies used subjective measures of PA, they did not show changes after intervention. This finding may suggest the superiority of objective measures of PA for identifying intervention-induced changes, consistent with prior reviews that advocate for standardized accelerometer evaluation in PA interventions [43] with young children. Objective measures may also be more suitable to capture the sporadic short bursts of movement, which tend to typify PA in early childhood and can be missed with subjective assessments. Nevertheless, objective measures do not capture PA and parenting styles, environmental conditions or stimuli, or PA behaviors and patterns of other family members, all of which can impact PA in young children.

Lastly, studies that addressed multiple outcome targets (ie, PA and nutrition) and modalities (websites + face-to-face interventions) did not have clear advantages. This is in contrast to a review of studies regarding the effects of mobile apps on health outcomes across age groups, which showed that multimodality studies had greater effects [44]. The lack of this documented advantage in our review may be due to the limited number of studies and the lack of high-quality studies without bias. Future intervention work should consider exploring the impact of individual intervention components versus the intervention as a whole. This would provide insights into how digital modalities are influencing or driving intervention outcomes.

The most significant findings of this review lie in highlighting opportunities for further work in this area related to the quality of the studies, intervention strategies, and inclusion of implementation outcomes. Except for 1 study, the rest had samples sizes less than 100; future work can expand the size and diversity of samples to understand the different settings and circumstances in which technology will work to increase PA in children (ie, evaluate mediators and moderators). Only 1 study assessed outcomes after 6 months postintervention. Future work should extend the time of follow-up used in prior studies, which have noted that technology-based intervention effects may not be long-lasting. Such a study is in progress that will examine the effects of a mobile app on children’s activity’s levels over 24 weeks [45].

Regarding the use of digital platforms as an intervention strategy for young children, prior studies have raised concerns that digital platforms may undermine traditional forms of exercise and contribute to excessive screen time in children [46]. Future work must examine if this is true. Conversely, young children who are active in using technology may increase activity in their daily routines (beyond technology time); this means technology time displaces other screen time rather than increasing it. These outcomes were not assessed in the studies presented in this report. Digital interventions have the potential to reach children and families with PA interventions in areas where structured, face-to-face programs are limited [23].

Combined with the inconsistent collection of implementation outcomes, studies failing to find effects may be unable to distinguish failure of the intervention from failure of the study design or from failure of implementation. Work beyond assessing acceptability (after feasibility is established) will be needed to provide better understanding of the implementation process for using technology to target young children for improving PA. As digitally based interventions are integrated into existing systems (eg, childcare, schools, and homes), the implementation outcomes missing in these studies will become particularly important (ie, adoption, cost, penetration, and sustainment). Further, future interventions could benefit from assessing the adoption rates by participant characteristics, costs of intervention delivery to inform future scalability, and sustained use of digital strategies beyond research study contexts [37].

The key limitation of this report is the lack of conclusive statements about digitally based PA interventions due to the emerging nature of the field. This is reflected in the low number and limited quality of prior studies in this area to date. Further, our review included only peer-reviewed full-text and English language publications; other unpublished and non-English research papers may exist on this topic. These factors limited the conclusions that could be drawn from this review. The strengths of our work include the systematic approach, use of the most updated guidelines on completion of systematic reviews; assessment of study quality, bias, and strength of the evidence; and high agreement levels between the reviewers.

### Conclusions

Across a range of physical health, psychological health, and cognitive development aspects, PA has been associated with positive outcomes for young children. Effective and scalable intervention methods are needed to help children achieve the recommended levels of PA and movement during their preschool years. This review suggests that interventions involving child-centered approaches may be the most promising for increasing PA in children. Specifically, commercial products packaged for delivery as PA interventions in schools used in 2 studies had significant effects on PA according to this review. This indicates opportunities to develop new digitally based interventions that are designed considering the needs of young users and their families. Although this review demonstrates that digital platforms are promising to a certain extent for achieving increased PA in children, there are numerous gaps in the current evidence. Further research using rigorous designs to achieve high study quality and minimize bias, monitoring implementation outcomes, and distinguishing the contributions of digital components from other intervention components will advance the understanding of the effectiveness of interventions and their potential to be implemented more broadly (versus those with characteristics limiting implementability). Additionally, future research focus should be on rigorous study designs involving diverse populations engaged in interventions delivered to the family and children that include objectively measured PA levels as primary or secondary outcomes.

## Appendix

Multimedia Appendix 1 Quality assessment of included studies.

Multimedia Appendix 2 Characteristics of included studies by global quality rating.

Multimedia Appendix 3 Implementation characteristics and outcomes of included studies by Global Quality Rating.

## Abbreviations

LPA: light physical activity
MVPA: moderate to vigorous physical activity
PA: physical activity
