# Supplementary material for: Digital Intervention Strategies for Increasing Physical Activity Among Preschoolers: Systematic Review
Source: J Med Internet Res. 2022 Jan 11;24(1):e28230. doi: 10.2196/28230 (PMC8790686; doi:10.2196/28230)
Supplement: Multimedia Appendix 1 [file jmir_v24i1e28230_app1.docx]

| **Table 1. Quality Assessment of Included Studies** | | | | | | | |
| --- | --- | --- | --- | --- | --- | --- | --- |
| **References** | **Selection Bias** | **Study Design** | **Confounders** | **Blinding** | **Data Collection Methods** | **Withdrawals and Dropouts** | **Global Rating** |
| Fu et al., (2018).^33^ | Strong | Moderate | Weak | Weak | Strong | Strong | Weak |
| Gao et al., (2019a).^30^ | Moderate | Weak | Moderate | Weak | Strong | Strong | Weak |
| Gao et al., (2019b).^35^ | Strong | Moderate | Strong | Weak | Strong | Strong | Moderate |
| Hammersley et al., (2019).^28^ | Strong | Strong | Strong | Moderate | Strong | Strong | Strong |
| Knowlden et al., (2015).^31^ | Strong | Strong | Strong | Moderate | Strong | Strong | Moderate |
| Ling et al., (2018).^34^ | Weak | Weak | Strong | Weak | Strong | Strong | Weak |
| Nyströmet al., (2017).^29^ | Moderate | Strong | Strong | Moderate | Strong | Strong | Strong |
| Sun et al., (2017).^32^ | Weak | Strong | Strong | Moderate | Moderate | Strong | Moderate |
