# Supplementary material for: Digital Intervention Strategies for Increasing Physical Activity Among Preschoolers: Systematic Review
Source: J Med Internet Res. 2022 Jan 11;24(1):e28230. doi: 10.2196/28230 (PMC8790686; doi:10.2196/28230)
Supplement: Multimedia Appendix 2 [file jmir_v24i1e28230_app2.docx]

| **Table 2. Characteristics of included studies by Global Quality Rating** | | | | | | | | |
| --- | --- | --- | --- | --- | --- | --- | --- | --- |
| **Study and Country** | **Study Design** | **Study Population** | **Modality** | **Exposure and Dose** | **Intervention Content** | **Measures** | **Data Analysis** | **Results** |
| **Strong Global Quality Rating** | | | | | | | | |
| Hammersley et al., (2019). Australia^28^ | RCT design | 86 parent-child dyads;  Children (M_age_ = 3.36 ± 0.80 yrs);  Intervention (n = 42), Control (n = 44).  6% Aboriginal; 92% not Aboriginal or Strait Islander | Website + email + social media (Facebook) directed at parent | Intervention group participated in an online health education lifestyle program for 11 weeks.  Comparison group received weekly emails and feedback. | - 6 modules on: introduction to the program, nutrition (2), physical activity, screen time, and sleep. - Quizzes - SMART goals setting with feedback from dietician - Weekly email reminders - Closed Facebook group that allowed participants to connect with others & dietician; incentivized to post module-relevant content | Preschooler: objectively-measured BMI, accelerometer-determined PA, sleep, food intake, and screen time.  Parents: child feeding, parent modeling, and nutrition self-efficacy. | Linear mixed models | Significant effects: food intake, child feeding, nutrition self-efficacy.  No effects: BMI, PA, sleep, screen time, and parent modeling. |
| Nyströmet al., (2017). Sweden^29^ | RCT design | 315 children (4.5 yrs);  Intervention (n = 156), Control (n = 159).  Parents in Ostergotland, Sweden | Mobile application targeted at parent | Intervention group participated in mobile-based, parent-directed intervention intended to stop obesity in preschool-aged children (MINISTOP) program for 6 months. Control group received a pamphlet on healthy eating and PA based on the existing guidelines for preschoolers. | - Freely accessed information, advice and evidence-based strategies on 12 themes: healthy foods, breakfast, small meals, physical activity and sedentary behavior, candy and sweets, fruits and vegetable, drinks, eating between meals, fast food, sleep, foods outside the home, foods at special occasions. - Regular push notifications. - Weekly graphic feedback on submitted information. - Option to ask questions of dietitian or psychologist. | Objectively-measured fat mass index, accelerometer- determined MVPA, sedentary behavior, and dietary (i.e., intakes of fruits, vegetables, candy, and sweetened beverages). | Wilcoxon’s rank-sum test | Significant effects: intakes of sweetened beverages, composite score of outcome measures.  No effects: fat mass index, MVPA, intakes of fruits, vegetables, and candy. |
| **Moderate Global Quality Rating** | | | | | | | | |
| Gao et al., (2019b). USA^35^ | RCT design | 32 preschoolers (M_age_ = 4.72 ± 0.73 yrs);  Intervention (n =18), Control (n =14).  56% Asian American, 32% White, 6% Black | Exergames (LeapTV console) directed at parent-child dyads | Intervention group participated in a home-based educational exergaming program 30 minutes daily, 5 days per week for 12 weeks.  Control group maintained regular PA patterns. | - Parents supported children to complete 5, 30-min sessions per week. - Calendar with daily duration goals for parent tracking. - TA call 2 days after LeapTV install. - TA visit (1 to 2) with first 2 weeks. - Monthly option to switch games. - Discouraged sedentary screen time. | Objectively-measured BMI, accelerometers-determined energy expenditure, cardiovascular fitness, and cognitive flexibility. | Analysis of covariance | Significant effect: cognitive flexibility  No effects:  BMI, energy expenditure, and cardiovascular fitness. |
| Knowlden et al., (2015). USA^31^ | RCT design | 57 mother-child dyads;  Children (4-6 yrs);  Intervention (n = 28), Control (n = 29).  67% White, 9% Black, 7% Asian, 12% Hispanic | Website directed at parents | Intervention group participated in the Enabling Mothers to Prevent Pediatric Obesity through Web-Based Education and Reciprocal Determinism (EMPOWER), a SCT-base program for 6 weeks.  Control group participated in a knowledge-based healthy lifestyle program. | - 5, 10-15 min audio visual presentations focused on child’s PA, fruit and vegetable intake, beverage consumption, screen time, nutrition and PA - Interactive worksheets - Discussion board posts | Preschooler:  parent-reported PA, fruit and vegetable consumption, sugar-free beverage consumption, and screen time.  Mothers: maternal-facilitated constructs of SCT, including environment, emotional coping, expectations, self-control, and self-efficacy. | Analysis of covariance; Multivariate analysis of variance | Significant effects: fruit and vegetable consumption, SCT constructs of environment.  No effects: PA, sugar-free beverage consumption, and screen time. |
| Sun et al., (2017). USA^32^ | Feasibility study with RCT design | 32 mother–child dyads;  Children (M_age_ = 4.31 ± 0.69 yrs);  Intervention (n = 16), Control (n = 16)  Chinese Americans in Northern California | Pre-loaded tablet with content directed at parents | Intervention group received weekly 30-minute, interactive, Cantonese, parent-directed educational modules delivered through a website via tablet computers for 8 weeks. Intervention based on InformationMotivation-Behavior model.  Control group received weekly mailings of printed health information relevant to child health behaviors. | - Culturally-adapted content for parents on 5-4-3-2-1-0, energy balance, “feeding my family,” grocery shopping, fun in PA, decreasing screen time, parenting, and healthy weight maintenance. - Modules included videos, animations, & songs. | Preschoolers: objectively-measured BMI, WC, parent-reported child eating and activity behaviors;  Mothers:  BMI, WC, pedometer-determined steps, eating behaviors, child-feeding behaviors, maternal self-efficacy. | Multilevel liner regression models | Significant effects:  maternal BMI, maternal WC, maternal eating behaviors and self-efficacy for promoting healthy eating.  No effects: child’s BMI, child WC, steps, eating and activity behaviors. |
| **Weak Global Quality Rating** | | | | | | | | |
| Fu et al., (2018). USA^33^ | RCT design | 65 preschoolers (M_age_ = 4.9±0.7 yrs);  Intervention (n =36), Control (n =29).  68% White, 17% Asian American, 11% Hispanic, 5% Black | Exergames  directed at children | Intervention group participated in a school-based exergaming program for 12 weeks.  Control group performed free play. | - Exergaming included in school routine 30 minutes daily, 5 times per week. - Games included GoNoodle (10 min), Adventures to Fitness (10 min), Cosmic Kids Yoga (5 min) | Pedometer-determined steps, gross motor development, and enjoyment of movement. | Multivariate analysis of variance | Significant effects: gross motor development, and  steps  No effect: enjoyment of movement |
| Gao (2019a) ^30^ | Quasi-experimental  design | 56 preschoolers (M_age_ = 4.46 ± 0.46 yrs);  Intervention (n =20), Control (n =36).  41% White, 30% Black, 16% Hispanic, 5% Asian | Exergames directed at children | Intervention group participated in a school-based exergaming program for 8 weeks.  Control group received usual care recess at school. | - 8 exergaming stations added to preschool - Offered 20 minutes daily, 5 days per week + warm up and cool down. - Research assistant/teacher ensured continuous play. | Accelerometers-determined MVPA, perceived competence, and motor skill competence. | Multivariate analysis of variance | Significant effect: MVPA.  No effects: perceived competence and motor skill competence. |
| Ling et al., (2018). USA^34^ | Feasibility study with quasi-experimental design | 69 parent-child dyads;  Children (M_age_ = 54.1 ± 6.2 months);  Intervention (n = 39), Control (n = 30).  36.2% White, 42% Black; 14.5% Hispanic | Social media (Facebook) directed at parents + in person meetings + preschool-based program directed at children | Intervention group participated in a lifestyle program for 10 weeks. Intervention based on Actor Partner Independence model.  Control group participated in the Head Start usual care activities. | - Support group with tips, health information, strategies, and family activities. - Weekly challenges and quiz. - Daily step target building to 10,000 for parents. - Progress wall recording steps, challenges, and quizzes. - 3 face-to-face meetings for connection, education, and discussion. Taste testing. - Received slow cooker, Garmin Vivofit2 - 40 sessions of 30 min activity at preschool (4 per week for 10 weeks). | Preschoolers’ objectively-measured BMIz, WC, accelerometer- determined MVPA; fruit/vegetable intake, screen time.  Parents: BMI, WC,  Accelerometer-determined MVPA, PA self-efficacy, PA knowledge, PA support, fruit/vegetable intake, nutrition knowledge, feeding skill, nutrition self-efficacy, nutrition support. | Linear mixed models | Significant effects: none. |
