# Supplementary material for: Digital Intervention Strategies for Increasing Physical Activity Among Preschoolers: Systematic Review
Source: J Med Internet Res. 2022 Jan 11;24(1):e28230. doi: 10.2196/28230 (PMC8790686; doi:10.2196/28230)
Supplement: Multimedia Appendix 3 [file jmir_v24i1e28230_app3.docx]

|  | | | | | |  |
| --- | --- | --- | --- | --- | --- | --- |
| **Table 3. Implementation Characteristics and Outcomes of Included Studies by Global Quality Rating** | | | | | | |
| **Study** | **Behavior Change Theory** | **Implementation Outcome(s)** | **Measure Used** | **Data Source** | **Results** | |
| **Strong Global Quality Rating** | | | | | | |
| Hammersley et al., (2019). Australia^28^ | Social cognitive theory | Acceptability  (with some questions on Appropriateness) | Self-developed survey | Users completed at the end of the intervention | Content and format received “high rate” of user acceptability | |
| Nyström et al., (2017). Sweden^29^ | Social cognitive theory | Acceptability | Number of feedback messages read; Number of recordings for fruits, candy, sweetened beverages, and sedentary behavior | Data from the MHealth platform | Unclear evidence to support evaluation of acceptability. | |
| **Moderate Global Quality Rating** | | | | | | |
| Gao et al., (2019b). USA^35^ | None | Fidelity | Self-developed checklist | Research team observation during home visits or phone interview | Researchers set a target of 90% for families and set goals if fidelity was below target. Overall observed fidelity not reported | |
| Knowlden et al., (2015). USA^31^ | Social cognitive theory | Fidelity | Self-developed survey | Users completed at the end of the intervention | Claimed that fidelity exceeded desired level for both groups. | |
| Sun et al., (2017). USA^32^ | Information-Motivation-Behavior model | Acceptability and (cultural) appropriateness | Feedback from mothers | 8 Chinese mothers in a focus group before the study; Mothers in phone interviews at end of study | Attributed positive perceptions of mothers to success of the intervention.  Also claimed the intervention was feasible without explicit measures or indicators of feasibility. | |
| **Weak Global Quality Rating** | | | | | | |
| Fu et al., (2018) USA^33^ | None | None | N/A | N/A | N/A | |
| Gao et al., (2019a). USA^30^ | None | Fidelity | Self-developed checklist | Research team observation of school lessons | Reported 90% fidelity to protocol as a process measure | |
| Ling et al., (2018). USA^34^ | Actor-Partner Interdependence Model | 1. Acceptability 2. Feasibility | 1. Self-developed survey for teachers and interview guide for caregivers. 2. (a) enrollment rates, (b) attrition rates, (c) attendance rates, and (d) challenge/quiz completion rates | 1. All teachers completed survey; 15 randomly selected caregivers interviewed at the end of the intervention. 2. Study records | 1. Means of 1.83 – 2.33 on 0 to 3 scale for items on teacher acceptability survey; length was least acceptable; ease of use, confidence to complete, satisfaction, and plans to continue use were high. All caregivers reported positive influence of the intervention. 2. (a) 37% (b) 1% (c) 77.3% for children; 48.7% of caregivers attended all sessions (d) 79.4% | |
| N/A = Not Applicable | | | | | | |
